# Complications and health-related quality of life after robot-assisted versus open radical cystectomy: a systematic review and meta-analysis of four RCTs

**DOI:** 10.1186/s13643-017-0547-y

**Published:** 2017-08-02

**Authors:** Susanne Vahr Lauridsen, Hanne Tønnesen, Bente Thoft Jensen, Bruno Neuner, Peter Thind, Thordis Thomsen

**Affiliations:** 1 Department of Urology, Copenhagen University Hospital, 2112, Rigshospitalet, 2100 Copenhagen, Denmark; 2 Clinical Health Promotion Centre, Bispebjerg and Frederiksberg Hospital, Copenhagen University Hospitals, Copenhagen, Denmark; 3 Clinical Health Promotion Centre, Health Sciences, Lund University, Lund, Sweden; 4 Health Science, University of Southern Denmark, Odense, Denmark; 5 Department of Urology, Aarhus University Hospital and Centre of Research in Rehabilitation, Aarhus University, Aarhus, Denmark; 6 Department of Anesthesiology and Intensive Care Medicine, Charité—Universitätsmedizin Berlin, Berlin, Germany; 7 Abdominal Centre, University Hospital of Copenhagen, Health and Medical Sciences, Rigshospitalet and University of Copenhagen, Copenhagen, Denmark

**Keywords:** Robot-assisted radical cystectomy, Open radical cystectomy, Postoperative complications, Health-related quality of life, Clavien-Dindo classification

## Abstract

**Background:**

Radical cystectomy is associated with high rates of perioperative morbidity. Robotic-assisted radical cystectomy (RARC) is widely used today despite limited evidence for clinical superiority. The aim of this review was to evaluate the effect of RARC compared to open radical cystectomy (ORC) on complications and secondary on length of stay, time back to work and health-related quality of life (HRQoL).

**Methods:**

The databases PubMed, The Cochrane Library, Embase and CINAHL were searched. A systematic review according to the PRISMA guidelines and cumulative analysis was conducted. Randomized controlled trials (RCTs) that examined RARC compared to ORC were included in this review. We assessed the quality of evidence using the Cochrane Collaboration’s ‘Risk of bias’ tool and Grading of Recommendations Assessment, Development and Evaluation approach. Data were extracted and analysed.

**Results:**

The search retrieved 273 articles. Four RCTs were included involving overall 239 patients. The quality of the evidence was of low to moderate quality. There was no significant difference between RARC and ORC in the number of patients developing complications within 30 or 90 days postoperatively or in overall grade 3–5 complications within 30 or 90 days postoperatively. Types of complications differed between the RARC and the ORC group. Likewise, length of stay and HRQoL at 3 and 6 months did not differ.

**Conclusion:**

Our review presents evidence for RARC not being superior to ORC regarding complications, LOS and HRQoL. High-quality studies with consistent registration of complications and patient-related outcomes are warranted.

**Systematic review registration:**

PROSPERO CRD42016038232

**Electronic supplementary material:**

The online version of this article (doi:10.1186/s13643-017-0547-y) contains supplementary material, which is available to authorized users.

## Background

Worldwide, bladder cancer is the ninth most common cancer with an estimated 429,800 new cases and 165,100 deaths in 2012. In the Western world, bladder cancer is the fourth and ninth most common cancer in men and women, respectively. Approximately, 30% of all newly diagnosed patients present with muscle-invasive bladder cancer (MIBC) [1, 2]. Radical cystectomy is the standard treatment for patients with muscle-invasive bladder cancer and in selected patients with non-muscle-invasive bladder cancer [1]. Patients undergoing radical cystectomy are at high risk of perioperative morbidity with about 60% experiencing at least one complication within 90 days after surgery [3, 4]. Open radical cystectomy (ORC) with pelvic lymph node dissection is considered the gold standard technique even though laparoscopic radical cystectomy (LRC) has been possible since 2001 [5]. In 2003, robot-assisted radical cystectomy (RARC) was introduced [6] and from 2004 to 2010, the utilization of RARC has increased from <1 to 13% [7].

Minimally invasive surgery may reduce the surgical stress response compared to open surgery [8], and RARC seems to be advantageous in eldery people with regard to complications [9]. In systematic reviews including both randomized controlled trials (RCTs), retrospective and prospective comparative study designs, RARC has similar oncological outcomes compared to ORC [10, 11], however with lower perioperative blood loss, fewer transfusions and shorter postoperative length of stay (LOS). Further, the reviews conclude that, in appropriately selected patients, RARC appears to be associated with significantly fewer total complications [4, 10, 12–14]. These results are not confirmed in randomized controlled trials comparing RARC with ORC [15–18].

Complications have traditionally been seen as a surrogate marker of quality in surgery [19], but little is known about how complications influence postoperative health-related quality of life (HRQoL). Today, including patient-related outcomes when evaluating new surgical techniques is therefore mandatory [20, 21]. If RARC reduces complications rates, it could be expected that patients undergoing RARC would have a shorter LOS and experience less negative impact postoperatively on HRQoL. The aim of this systematic review was to evaluate the evidence from RCTs of robot-assisted radical cystectomy (RARC) versus open radical cystectomy (ORC) in regard to primarily complications, and secondly LOS, HRQoL and time back to work or habitual activity.

## Methods

### Protocol

Analysis methods and inclusion criteria for this systematic review and meta-analysis were specified in advance and documented in a protocol in compliance with the ‘Preferred Reporting Items for Systematic Reviews and Meta-Analyses’ (PRISMA) Statement [22] (See Additional file 1). The protocol was registered with the PROSPERO database in April 2016 (CRD42016038232).

The primary outcome was the number of patients with postoperative complications requiring treatment within 30 and 90 days. Complication rates were calculated using the total number of patients randomized to ORC and RARC respectively as the denominator. Secondary outcomes were total number of postoperative complications within 30 and 90 days, type of complications, LOS, time back to work or habitual activity and HRQoL as measured by validated disease specific and/or generic scales. Due to inconsistency in reporting of complications, it was not possible to perform a meta-analysis of total number of complications. Instead, we performed a meta-analysis of grade 3–5 complications.

### Search strategy

The databases PubMed, The Cochrane Library, Embase and CINAHL were initially searched August 2015 using the following search terms and strategy: bladder cancer, open radical cystectomy, robot-assisted radical cystectomy, postoperative complications, intraoperative complications, postoperative pulmonary complications, postoperative cardiovascular complications, postoperative wound complication, postoperative morbidity, postoperative mortality, postoperative quality of life, postoperative length of stay, postoperative time back to work and postoperative cancer relapse (see Additional file 2).

The search was limited to patients aged 18 years or more. No language or date limits were applied. A full up-date of the searches was done September 2016.

Clinicaltrials.gov was searched to identify ongoing and unpublished studies. Studies were checked for additional relevant citations.

### Criteria for considering studies for this review

We included RCTs comparing RARC to ORC and reporting at least one outcome of interest. The reconstruction method for urinary diversion should preferably be described as extra-corporeal or intra-corporeal.

### Definition of complications

To compare complications across studies in a systematic, objective and reproducible way, it is recommended to use a standardized classification [19]. We defined a postoperative complication as any complications needing treatment in accordance with the “Clavien-Dindo” classification [23].

### Data extraction

Two authors (BTJ and SVL) reviewed all records retrieved from the search and included studies according to the inclusion criteria. Discrepancies were resolved by discussion. BTJ and SVL individually extracted data. Discrepancies were solved by PT. We extracted the following study characteristics from the included studies: author, country, year of publication, number of participants, types of surgery (ORC, RARC), intra-corporal or extra-corporal urinary diversion, inclusion and exclusion criteria, degree of follow-up and definition of complications.

Furthermore, we extracted data on age, body mass index (BMI), Charlsons comorbidity index (CCI) [24], American Society of Anesthesiologists physical status classification system (ASA) [25], gender, smoker, tumour (pT and pN), total lymph nodes retrieved, surgical margins, type of urinary diversion, neoadjuvant chemotherapy, complication rates within 30 days or 90 days post-operatively, types of complications, length of stay, HRQoL and time back to work/daily activity.

### Assessment of reporting of complications

To assess the quality of reporting of complications after urologic procedures using the Clavien-Dindo classification, we used the data extraction form from the European Association of Urology guideline “Reporting and Grading of Complications after Urologic Surgical Procedures” [19]. This form evaluates the number of Martin et al. criteria for accurate and comprehensive reporting of surgical complications [26] and the use of Clavien-Dindo classification of complications. The Clavien-Dindo classification includes five grades of complications based on the main criterion of the intervention needed to resolve the complication.

### Risk of bias and quality assessment

Risk of bias was assessed using the Cochrane Collaboration’s tool for assessing risk of bias [27]. This involved assessment of sequence generation, allocation concealment, blinding of participants, personal and outcome, incomplete outcome data, selective outcome reporting and other sources of bias. The Grading of Recommendations Assessment, Development and Evaluation (GRADE) [28] approach was used to assess the quality of the evidence. BTJ and SVL individually assessed risk of bias and quality of evidence. Disagreements were resolved by TT.

### Statistical analysis

For purposes of analysis, robot-assisted radical cystectomy was considered the experimental group.

Cumulative analysis was conducted using Review Manager (RevMan) [Computer Programme]. Version 5.3. Copenhagen: The Nordic Cochrane Center, The Cochrane Collaboration, 2014. Statistical heterogeneity was calculated using the *I* ^2^ statistic, which describes the percentage of the variability in effect estimates that is due to heterogeneity rather than sampling error.

For dichotomous outcomes, results were calculated with the Mantel-Haenszel square method using the ‘fixed-effects’ meta-analytical technique to calculate risk ratios (RR) and corresponding 95% confidence intervals (CI). For continuous outcomes, the results were reported as mean differences (MD) and corresponding 95% CIs. Authors of the included studies were contacted for additional information in case of missing data.

## Results

**Fig. 1:**
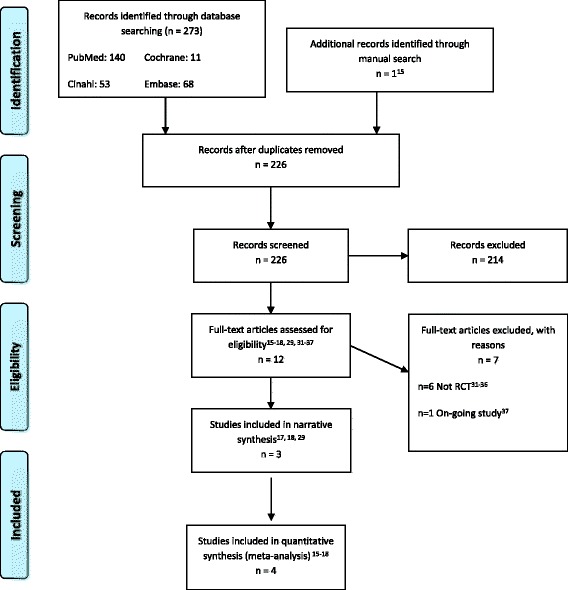
PRISMA flow-chart literature search

The literature search retrieved 273 records. Twelve articles [15–18, 29–35] met the criteria for considering studies. Of these, we included five articles covering four studies (Fig. 1) [15–18]. Two articles [16, 29] were based on the same trial but reported different outcomes: one reported complications and one HRQoL.

### Characteristics of included studies

Characteristics of included studies are summarized in Table 1. The two reviewers who extracted data were in total agreement. A total of 239 patient cases were analysed. Of these, 118 underwent ORC and 121 underwent RARC. Approximately 80% of patients were male. No studies reported the CCI score. ASA scores across studies were comparable with the majority of patients having ASA score 3. In Khan et al. [18], 75% of patients had an ASA score of 2. In the RARC group, 70% of patients presented with tumour stage T2 or lower compared to 63% in the ORC group. One study did not report tumour stage [16]. Parekh et al. [16] did not describe the type of urinary diversion performed; the remaining studies [15, 17, 18] performed extra-corporeal urinary diversion (Table 1). No studies described adverse events related to the surgical technique.

**Table 1:** Individual study characteristics

Reference	No (ORC/RARC)	Urinary diversion method	Exclusion criteria	Age, years (ORC/RARC)	Mean (%)	Matching outcomes
Nix et al. 2010 [15] USA	20/21	Extra-corporeal	Not surgical candidates Not allowing randomization Preconceived preference for ORC or RARC	69.2/67.4 (mean)	75.6	1,3,6
Parekh et al.^a^ 2013 [16] USA	20/20	Unclear	Inability to give informed consent, Multiple prior abd and pelvic surgery Morbid obesity Clinical T4 BC LN positive BC or retroperitoneal LN Preexisting condition precluding safe pneumoperitoneum Age <30 or >90 Pregnancy	64.5/69.5 (median)	85	1,3,6
Messer et al.^a^ 2014 [29] USA						5
Bochner et al. 2015 [17] USA	58/60	Extra-corporeal	Contraindication for Trendelenberg Extensive prior abd surgery	65/ 66 (median)	78.8	2,3,5,7
Khan et al. 2016 [18] UK	20/20	Extra-corporeal	Previous pelvic radiation T4 or M1 Contraindication for Trendelenberg Extensive prior abd surgery	66.6/68.6 (mean)	87.5	1,2,3,5,6,7

Outcomes: 1: number of patients with complications within 30 days, 2: number of patients with complications within 90 days, 3: length of stay (days), 4: time back to work, 5: quality of life, 6: number of grade 3–5 complications within 30 days, 7: number of grade 3–5 complications within 90 days

^a^Same population in Parekh and Messer

### Characteristics of excluded studies

Of the potentially eligible studies, six were excluded, as they were not RCTs; one study was a protocol for an on-going study. Aboumarzouk et al. [31] compared 155 patients undergoing LRC or ORC retrospectively. Aboumohamed et al. [30] reported retrospective data from patients undergoing radical cystectomy. Patients were grouped based on surgical approach (ORC vs RARC) and urinary diversion technique (extra-corporeal vs intra-corporeal). Atmaca et al. [32] retrospectively compared 42 open versus 32 intra-corporeal RARCs. Kahn et al. [33] reported data from a prospective cohort study of 158 patients from 2003 to 2008 undergoing ORC, LRC or RARC. Ng et al. [34] used a prospective cohort design including 187 consecutive patients undergoing either ORC or RARC. Finally, Niegisch et al. [35] prospectively collected data on 64 patients undergoing RARC and retrospectively compared these with 79 patients undergoing ORC. The on-going RAZOR (randomized open vs robotic cystectomy) trial [36] is a multi-institutional randomized clinical trial planning to enrol at least 320 patients from 15 different institutions. The aim of the RAZOR trial is to compare ORC with RARC, pelvic lymph node dissection (PLND) and urinary diversion in regard to oncological outcomes, complications and HRQoL measures with a primary endpoint of 2-year progression-free survival.

### Assessment of risk of bias

**Fig. 2:**
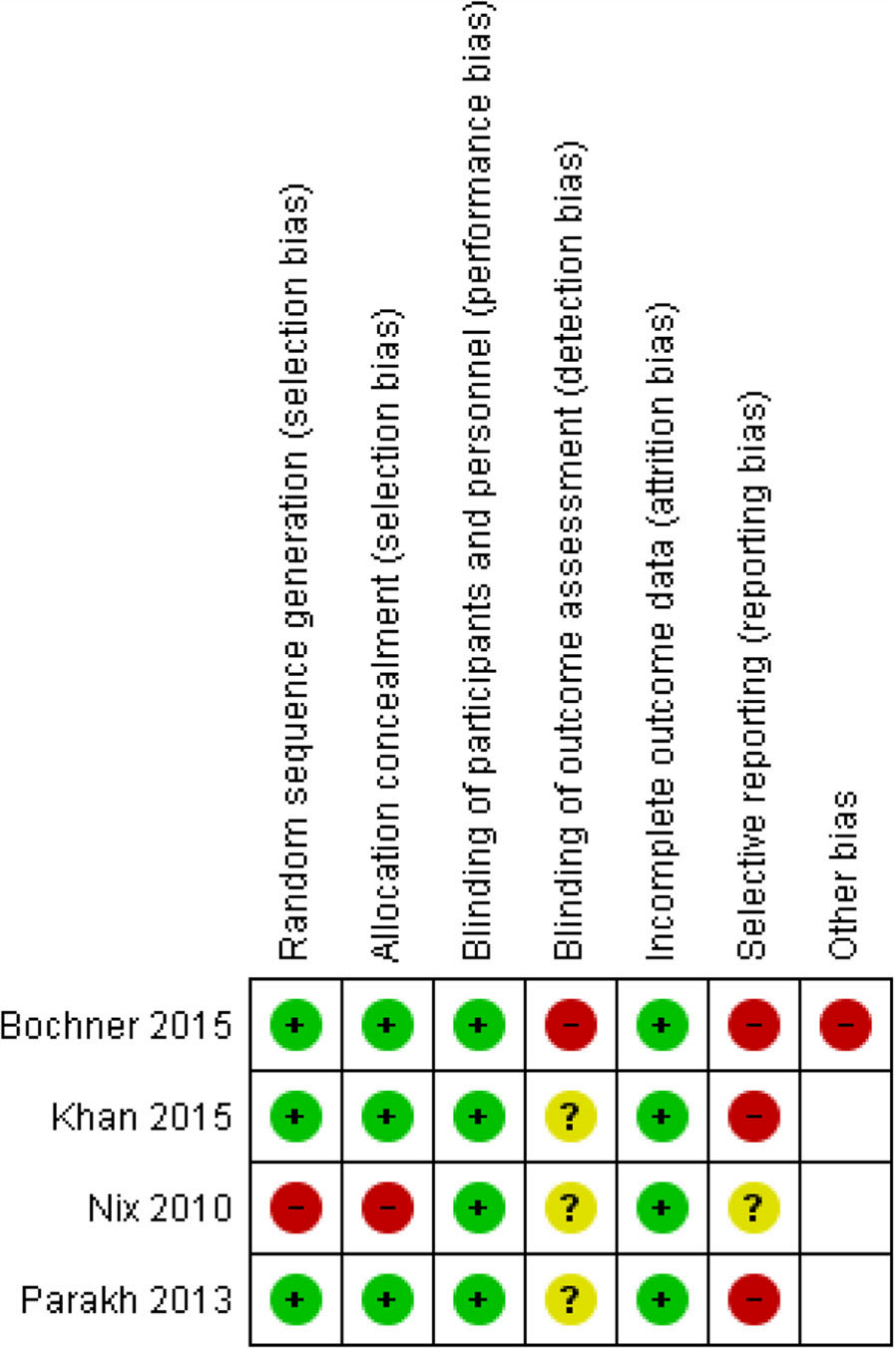
Risk of bias within studies

Overall, the studies were assessed to be at moderate risk of bias (Fig. 2). None of the studies had blinding of participants or personnel. We consider it unlikely that this influenced the primary outcome: perioperative complications. Therefore, the studies were assessed at low risk of performance bias. Random sequence generation and allocation concealment were assessed at high risk of bias in Nix et al. [15] because the randomisation schema was performed with five sequential patients undergoing one surgical approach before subsequently altering surgical technique. The study by Bochner et al. was assessed to be at high risk of detection bias due to non-blinding of outcome assessors [17]. The remaining studies did not report if outcome assessors were blinded and were therefore assessed to be at unclear risk of detection bias. Outcome data were incomplete in Bochner et al. [17]. Bochner et al. reported outcome data on quality of life from 23 of 60 patients in the RARC group and 34 of 58 patients in the ORC group. Selective outcome reporting was identified in Khan et al. [18] and Parekh et al. [16] with both reporting different outcomes in the trial protocol and final article. Other biases detected were incomplete reporting of complications by Bochner et al. [17] and Parekh et al. [16] and unclear reporting of complications in Nix et al. [15].

According to the GRADE assessment, the quality of the evidence for complications was low and for LOS, moderate (Table 2).

**Table 2:** Summary of findings

Quality assessment							No. of patients		Effect		Quality	Importance
No. of studies	Study design	Risk of bias	Inconsistency	Indirectness	Imprecision	Other considerations	Open	Robot-assisted radical cystectomy	Relative (95% CI)	Absolute (95% CI)		
Number of patients with complications within 90 days												
2	Randomized trials	Serious^a^	Not serious	Not serious	Serious^b^	None	48/80 (60.0%)	52/78 (66.7%)	RR 0.90 (0.71 to 1.14)	67 fewer per 1.000 (from 93 more to 193 fewer)	⨁⨁◯◯ Low	
Grade 3–5 complications 90 days (total complications grade 2–5)												
2	Randomized trials	Serious^a^	Not serious	Not serious	Serious^b^	None	22/58 (37.9%)	18/51 (35.3%)	RR 1.04 (0.64 to 1.71)	14 more per 1.000 (from 127 fewer to 251 more)	⨁⨁◯◯ Low	
Number of grade 3–5 complications within 30 days												
3	Randomized trials	Serious^a^	Not serious	Not serious	Serious^c^	None	13/28 (46.4%)	11/23 (47.8%)	RR 1.07 (0.61 to 1.87)	33 more per 1.000 (from 187 fewer to 416 more)	⨁⨁◯◯ Low	
Length of stay												
3	Randomized trials	Not serious	Not serious	Not serious	Serious^d^	None	100	98	–	MD 0.2 lower (1.54 lower to 1.14 higher)	⨁⨁⨁◯ Moderate	
Number of patients with complications within 30 days												
3	Randomized trials	Serious^a^	Not serious	Not serious	Serious^c^	None	23/61 (37.7%)	29/60 (48.3%)	RR 0.78 (0.53 to 1.16)	106 fewer per 1.000 (from 77 more to 227 fewer)	⨁⨁◯◯ Low	
Time back to work or habitual activity												
0							0	0	–	see comment	–	

*CI* confidence interval, *RR* risk ratio, *MD* mean difference

^a^Incomplete reporting of complications according to the Clavien-Dindo Classification

^b^Small sample size in one of the studies

^c^Number of events and sample sizes are very small

^d^Small sample sizes in two of the studies

### Complications

Three studies [15, 16, 18] classified complications according to the Clavien-Dindo classification [37], and one study [17] used the Memorial Sloan Kettering Cancer Centre (MSKCC) modified Clavien-Dindo classification [3], making comparison feasible. Reporting of complications was overall poor. One study [18] met eight of the ten Martin criteria, and three studies [15–17] met five of the ten Martin criteria. None of the included studies included blood transfusion as a complication even though this is a grade 2 complication according to the Clavien-Dindo classification. One study [16] reported blood transfusions separately. Two studies [16, 17] only reported grade 2–5 complications. Khan et al. [18] reported grade 1–5 complications, and it is unclear if all grades were assessed in Nix et al. [15] as they only reported median and mean values for the Clavien-Dindo units.

**Fig. 3:**
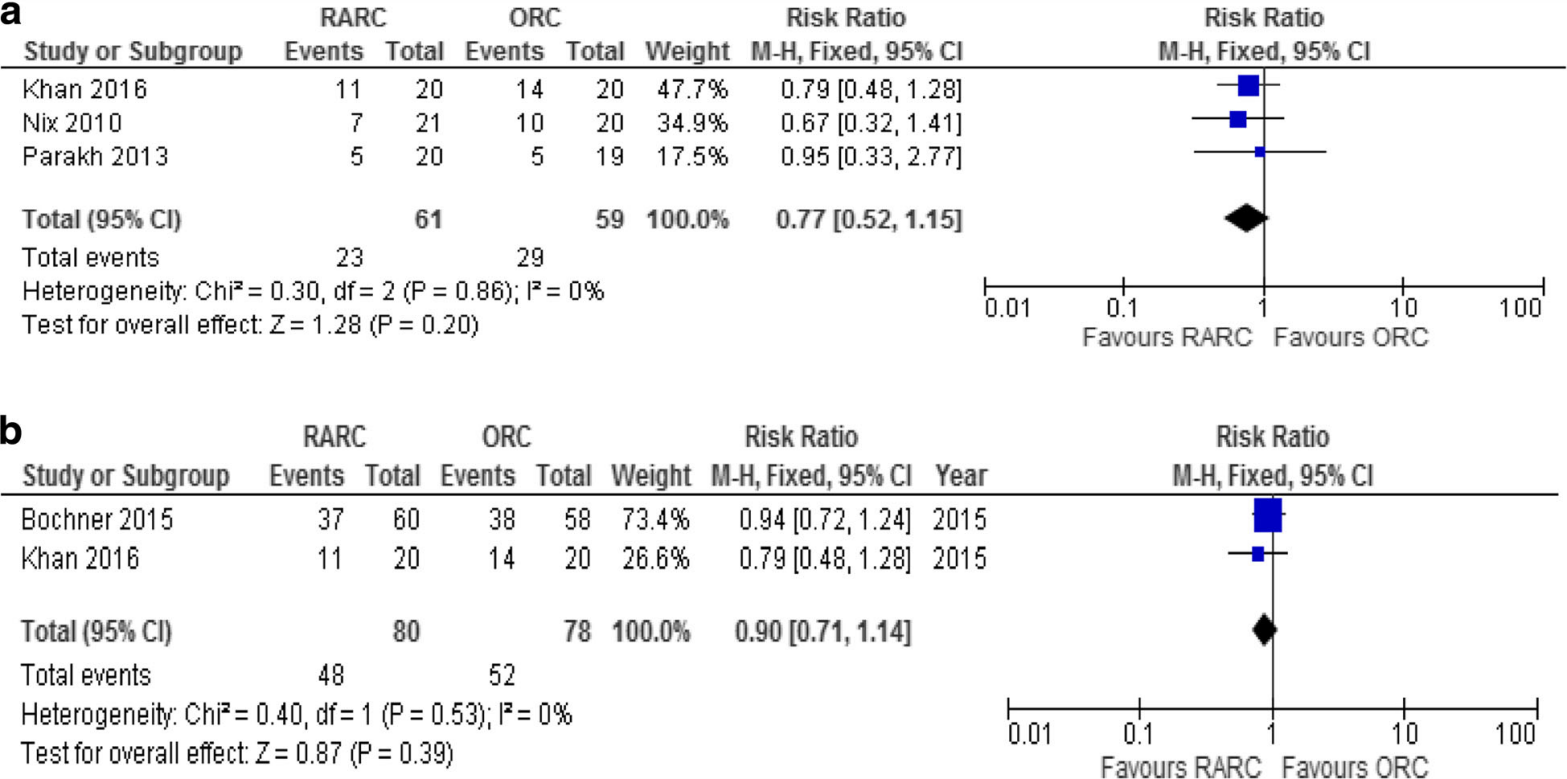
Forest plot. Number of patients with complications within **a** 30 and **b** 90 days

The number of patients with complications within 30 days postoperatively ranged from 20 to 55% in the RARC group and from 20 to 70% in the ORC group and, within 90 days postoperatively, from 55 to 62% in the RARC group and from 66 to 70% in the ORC group. Review Manager only allows analysis of continuous outcomes using means and standard deviations (SD). As Parekh only reported the median, we used the calculated mean and SD from the RARC Pasadena Consensus Panel Review [4] in the meta-analysis of LOS. The meta-analysis showed no statistically significant difference between RARC and ORC in the number of patients developing complications within 30 days postoperatively, RR 0.78 (95% CI, 0.53–1.16; *p* = 0.22) (Fig. 3 and Table 2). Complication rates within 90 days postoperatively similarly did not differ, RR 0.90 (95% CI, 0.71–1.14; *p* = 0.39) (Fig. 3 and Table 2). The quality of the evidence was low (Table 2).

#### Total number of postoperative complications within 30 days

**Fig. 4:**
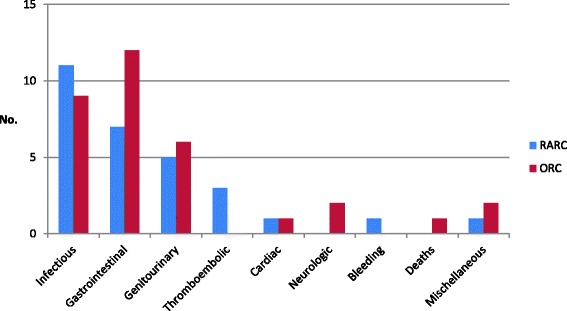
Type of complications within 30 days

Three studies [15, 16, 18] reported the total number of complications within 30 days. In the RARC group, a total of 23 events in 61 patients occurred and in the ORC group, 29 events in 60 patients. Types of complications differed between the two groups with infections and thromboembolic complications occurring more frequently in the RARC group (Fig. 4). Sub-group analyses according to types of complications showed no significant differences. Infections: RR 1.36 (95% CI, 0.69–2.70; *p* = 0.38); thromboembolic complications: RR 2.83 (95% CI, 0.49–16.50; *p =* 0.25). There were fewer gastrointestinal complications in the RARC group but not significantly RR 0.50 (95% CI, 0.22–1.16; *p* = 0.11. Miscellaneous complications in the open group included diagnosis of leukaemia and dehydration and, in the robotic group, evisceration.

**Fig. 5:**
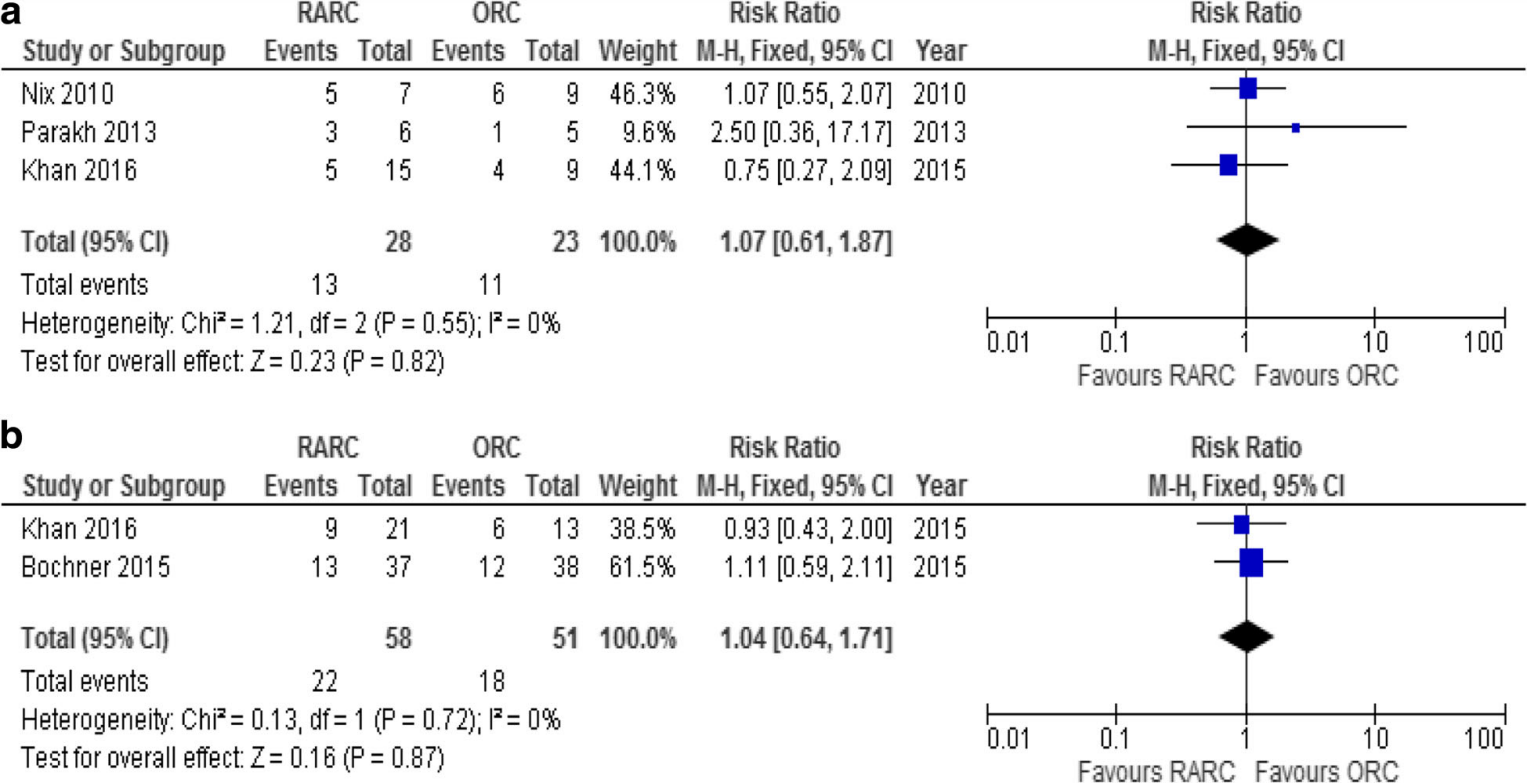
Forest plot. Grade 2–5 complications within **a** 30 and **b** 90 days

Meta-analysis of grade 1–2 complications was not possible due to inconsistency in reporting. Parekh et al. [16] only reported grade 2–5 complications. Meta-analysis of grade 3–5 complications within 30 days postoperatively showed no statistical difference between RARC and ORC, RR 1.07 (95% CI,0.61–1.87; *p* = 0.82) (Fig. 5).

#### Total number of postoperative complications within 90 days

**Fig. 6:**
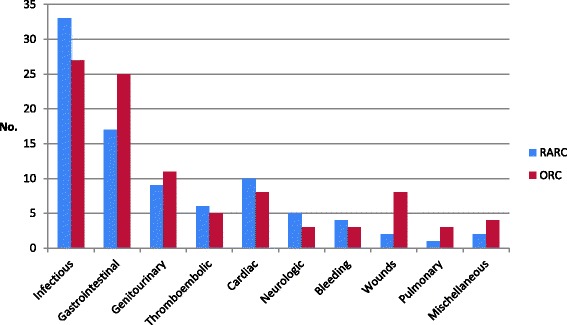
Type of complications within 90 days

Two studies [17, 18] reported complications within 90 days postoperatively. In the RARC group, a total of 89 events occurred in 80 patients and, in the ORC group, 97 events in 78 patients. Types of complications differed less within 90 days postoperatively and still sub-group analyses showed no significant differences. More infections were seen in the RARC group; RR 1.32 (95% CI 0.87–1.99; *p* = 0.19) as well as more cardiac complications; RR 1.21 (95% CI 0.54–2.72; *p =* 0.65). There were fewer gastrointestinal complications in the RARC group; RR 0.74 (95% CI 0.43–1.28; *p* = 0.28) and wound complications occurred: RR 0.26 (95% CI 0.06–1.13; *p* = 0.07). Miscellaneous complications were not specified [17] (Fig. 6). Bochner et al. [17] only reported grade 2–5 complications. Meta-analysis of grade 3–5 complications within 90 days showed no statistical difference between RARC and ORC (Fig. 5). RR 1.04 (95% CI 0.64–1.71; *p =* 0.87).

### Length of stay

**Fig. 7:**
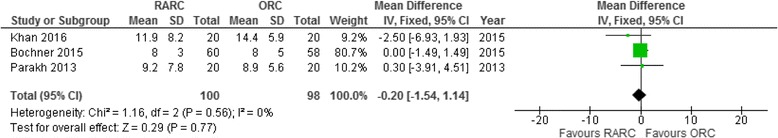
Forest plot. Length of stay (days)

Three studies with 198 patients reported data on LOS, measured in days. Pooled estimates showed no statistical difference between RARC and ORC, mean difference (MD) −0.20 (95% CI −1.54, 1.14; *p* = 0.77) (Fig. 7). The quality of the evidence was moderate (Table 2).

### Time back to work

No studies assessed this outcome.

### Quality of life

Three studies [17, 18, 29] assessed HRQoL in overall 198 patients. Khan et al. [18] measured HRQoL at 8 months postoperatively; Bochner et al. [17] at baseline and after 3 and 6 months; Messer et al. [29] at baseline, 3, 6, 9 and 12 months. Data on HRQoL were available from 114 of 198 patients (57.6%). Khan et al. [18] measured HRQoL using the Functional Assessment of Cancer Therapy-Bladder (FACT-Bl) and the Functional Assessment of Cancer-General (FACT-G) scale [38], Messer et al. [29] used the Functional Assessment of Cancer Therapy-Vanderbilt Cystectomy index scale [39]; Bochner et al. [17] used EORTC Quality of Life Questionnaire Core 30 (QLQ-C30) [40]. The FACT-G and QLQ-C30 scales are both cancer generic instruments, they are internationally validated and there is almost complete agreement between the two instruments in the nomenclature of the most important domains. However, when trying to convert FACT-G scores to QLQ-C30 scores, the social domain shows serious inconsistencies and is therefore not eligible for equating [41]. For this reason, we refrained from meta-analysis of the HRQoL outcome.

All three studies [17, 18, 29] assessing HLQoL found no significant differences between RARC and ORC at 3 and 6 months with the exception of Messer et al. [29], who found a 2.5-point lower score (FACT-VCI) in the ORC group for physical well-being at 6 months. This difference is not considered clinically relevant [29].

## Discussion

We hypothesised that RARC would reduce postoperative complications, LOS, time back to work and mitigate any negative impact of surgery on postoperative HRQoL [4, 11–13]. Based on data from four RCTs comparing RARC to ORC, robot-assisted radical cystectomy did not reduce the rate of postoperative complications or LOS in patients with bladder cancer. Likewise, postoperative HRQoL appeared to be similar in patients undergoing RARC and ORC. Time back to work was not assessed.

While the overall complication rates within 30 days postoperatively resemble the rates reported in the review by Novara et al. [4], their analyses showed a slightly lower rate for any grade and grade 3 complications within 90 days in favour of RARC. The absence of a difference in postoperative complications between RARC and ORC in this review may be explained by the inclusion of RCTs only, while former reviews [10–13] also included studies comparing prospective patients undergoing RARC to retrospective ORC data. The quality of the evidence in the present review was assessed to be low for the primary outcome, the number of patients developing complications postoperatively. Despite this, we consider that our review contributes relevantly to the evolving body of evidence within RARC, explicitly due to the exclusive inclusion of RCTs.

In this review, all studies used the Clavien-Dindo classification of complications. Still comparison was difficult because of unclear or incomplete reporting of complications. Judgement of complication status would also to some degree have been subjective, with a risk of intra-observer and inter-observer variation [42]. These factors hamper comparison of complication rates between studies even when the same classification is used and the strength of the conclusions that can be drawn from this review.

Radical cystectomy is a complex procedure, and the surgical technique per se may not influence the risk of postoperative complications as much as other identified predictors. Former reviews have for example identified ASA score and age at surgery as non-modifiable predictors for grade 3–5 complications [3, 4, 43]. In this review, the mean or median age was 66–69 in the RARC group and 65–69 in the ORC group. ASA scores were also comparable across the RARC and ORC groups, which may partially explain the similar postoperative complication rates in the two groups.

In this systematic review, all urinary diversions were performed extra-corporeally (ECUD) which may have influenced the outcomes and diminished the advantages of the robotic technique. This may be ascribed to the fact that the surgical stress response associated with extra-corporeal diversion is similar to the surgical stress response caused by an open approach [21]. Two studies [44, 45] comparing postoperative complications in patients undergoing ECUD and intra-corporeal urinary diversion (ICUD) found a trend in favour of ICUD. Ahmed et al. [44] compared 768 patients who had ECUD to 167 patients who had ICUD and found no statistically significant difference in complication rates within 30 days (43% in the ECUD group vs 35% in the ICUD group; *p* = 0.07). The authors estimated that about 18% of patients undergoing RARC had ICUD performed [44].

Several reviews [4, 11–13] have reported shorter LOS after RARC compared to ORC. This may be attributed to their findings of fewer complications in the RARC group. In this review, we did not identify a significant difference in LOS, possibly reflecting the identical complication rates in the RARC and in the ORC groups. Mean LOS ranged from 5 to 12 days in the robotic group and from 6 to 14 in the open group. The longest LOS was seen in the most recent study [18] and may be explained by different discharge criteria more than from surgical technique.

To the best of our knowledge, this is the first systematic review addressing both the inconsistencies in reporting of complications in studies comparing RARC and ORC and the quality of the evidence according to the GRADE criteria and the potential limitations this consequently infers on the conclusions that can be drawn. We found no differences between the RARC and ORC groups in complications, LOS and HRQoL at 3 or 6 months. Quality of life is a key component of the value of any treatment and should be considered in discussions when a new surgical technique is implemented. At present, we have insufficient data on HRQoL following RARC and ORC to determine whether RARC may be superior to ORC in regard to this outcome. Results from the RAZOR study [36] may give new insight in this field. Likewise, we lack data on time back to work or habitual activity as none of the studies addressed these outcomes. During the submission process, two additional reviews of the same four RCTs were published [46, 47]. Both reviews found results for perioperative complications similar to ours. Moreover, they found evidence for significantly reduced perioperative blood loss and a longer operating time in the RARC group.

### Limitations and strengths

A major limitation of this review is the few RCTs and cases. Furthermore, included studies were small; however, they had a relatively high frequency of complications. Only two studies [17, 18] reported power calculations for detecting clinically relevant differences in postoperative complications between the RARC and ORC groups. The lack of statistical power impedes firm conclusions regarding the potential superiority of RARC to ORC. The incomplete reporting of complications is a limitation. Future studies should observe guidelines for assessing and reporting of complications, for example the EAU guideline “Guidelines on Reporting and Grading of Complications after Urologic Surgical Procedures” [19] to ensure standardized, uniform and valid data acquisition. The inclusion of only RCTs and the absence of statistical heterogeneity between studies strengthen the conclusions that can be drawn from this review.

## Conclusion

Based on low to moderate quality evidence from four RCTs at moderate risk of bias, patients with bladder cancer undergoing RARC did not develop fewer complications or have shorter length of stay compared to patients undergoing ORC. There is a need for high-quality studies with consistent registration of complications according to guidelines. Knowledge of patients’ experience of HRQoL postoperatively and time back to work or habitual activity is warranted as there is a sparsity of evidence for these outcomes after RARC and ORC.

## Additional files

Additional file 1: PRISMA checklist. (DOC 62 kb)

Additional file 2: PubMed search. (DOCX 22 kb)

## Abbreviations

ASA: American Society of Anesthesiologists physical status classification system
BMI: Body mass index
CCI: Charlsons comorbidity index
CI: Confidence interval
ECUD: Extra-corporeal urinary diversion
EORTC: European Organisation for Research and Treatment of Cancer
FACT-B1: Functional Assessment of Cancer Therapy-Bladder
FACT-G: Functional Assessment of Cancer-General
FACT-VCI: Functional Assessment of Cancer Therapy-Vanderbilt Cystectomy Index scale
GRADE: Grading of Recommendations Assessment, Development and Evaluation
HRQoL: Health-related quality of life
ICUD: Intra-Corporeal urinary diversion
LOS: Length of stay
LRC: Laparoscopic radical cystectomy
MD: Mean difference
MIBC: Muscle invasive bladder cancer
MSKCC: Memorial Sloan Kettering Cancer Center
ORC: Open radical cystectomy
PLND: Pelvic lymph node dissection
QLQ-C30: Quality of Life Questionnaire Core 30
RARC: Robot-assisted radical cystectomy
RCT: Randomized controlled trial
RR: Risk ratios
SD: Standard deviation
