# Supplementary material for: Complications and health-related quality of life after robot-assisted versus open radical cystectomy: a systematic review and meta-analysis of four RCTs
Source: Syst Rev. 2017 Aug 2;6:150. doi: 10.1186/s13643-017-0547-y (PMC5541663; doi:10.1186/s13643-017-0547-y)
Supplement: Supplementary file 2 — PubMed search. (DOCX 22 kb) [file 13643_2017_547_MOESM2_ESM.docx]

**History**

[Download history](http://www.ncbi.nlm.nih.gov/pubmed?p$l=Email&Mode=download&dlid=history&filename=history.csv&db=pubmed&historyid=NCID_1_39735236_130.14.22.76_5555_1440508845_1340045005_0MetA0_S_HStore&p$debugoutput=off)[Clear history](http://www.ncbi.nlm.nih.gov/pubmed/advanced)

| Recent queries | | | | |
| --- | --- | --- | --- | --- |
| **Search** | **Add to builder** | **Query** | **Items found** | **Time** |
| [#17](http://www.ncbi.nlm.nih.gov/pubmed/advanced) | [Add](http://www.ncbi.nlm.nih.gov/pubmed/advanced) | Search **(((bladder cancer) AND open radical cystectomy) AND robot-assisted radical cystectomy) AND (((((((((((postoperative complications) OR intraoperative complication*) OR postoperative pulmonary complication*) OR postoperative cardiovascular complication*) OR postoperative wound complication*) OR postoperative morbidity) OR postoperative mortality) OR postoperative quality of life) OR postoperative length of stay) OR postoperative time back to work) OR postoperative cancer relapse)** | [48](http://www.ncbi.nlm.nih.gov/pubmed/?cmd=HistorySearch&querykey=17) | 09:30:11 |
| [#16](http://www.ncbi.nlm.nih.gov/pubmed/advanced) | [Add](http://www.ncbi.nlm.nih.gov/pubmed/advanced) | Search **(((bladder cancer) AND open radical cycstectomy) AND robot-assisted radical cystectomy) AND (((((((((((postoperative complications) OR intraoperative complication*) OR postoperative pulmonary complication*) OR postoperative cardiovascular complication*) OR postoperative wound complication*) OR postoperative morbidity) OR postoperative mortality) OR postoperative quality of life) OR postoperative length of stay) OR postoperative time back to work) OR postoperative cancer relapse)** | [48](http://www.ncbi.nlm.nih.gov/pubmed/?cmd=HistorySearch&querykey=16) | 09:30:10 |
| [#15](http://www.ncbi.nlm.nih.gov/pubmed/advanced) | [Add](http://www.ncbi.nlm.nih.gov/pubmed/advanced) | Search **((((((((((postoperative complications) OR intraoperative complication*) OR postoperative pulmonary complication*) OR postoperative cardiovascular complication*) OR postoperative wound complication*) OR postoperative morbidity) OR postoperative mortality) OR postoperative quality of life) OR postoperative length of stay) OR postoperative time back to work) OR postoperative cancer relapse** | [584393](http://www.ncbi.nlm.nih.gov/pubmed/?cmd=HistorySearch&querykey=15) | 09:29:23 |
| [#14](http://www.ncbi.nlm.nih.gov/pubmed/advanced) | [Add](http://www.ncbi.nlm.nih.gov/pubmed/advanced) | Search **postoperative cancer relapse** | [29578](http://www.ncbi.nlm.nih.gov/pubmed/?cmd=HistorySearch&querykey=14) | 09:27:16 |
| [#13](http://www.ncbi.nlm.nih.gov/pubmed/advanced) | [Add](http://www.ncbi.nlm.nih.gov/pubmed/advanced) | Search **postoperative time back to work** | [1124](http://www.ncbi.nlm.nih.gov/pubmed/?cmd=HistorySearch&querykey=13) | 09:26:51 |
| [#12](http://www.ncbi.nlm.nih.gov/pubmed/advanced) | [Add](http://www.ncbi.nlm.nih.gov/pubmed/advanced) | Search **postoperative length of stay** | [27083](http://www.ncbi.nlm.nih.gov/pubmed/?cmd=HistorySearch&querykey=12) | 09:26:23 |
| [#11](http://www.ncbi.nlm.nih.gov/pubmed/advanced) | [Add](http://www.ncbi.nlm.nih.gov/pubmed/advanced) | Search **postoperative quality of life** | [15932](http://www.ncbi.nlm.nih.gov/pubmed/?cmd=HistorySearch&querykey=11) | 09:26:00 |
| [#10](http://www.ncbi.nlm.nih.gov/pubmed/advanced) | [Add](http://www.ncbi.nlm.nih.gov/pubmed/advanced) | Search **postoperative mortality** | [93935](http://www.ncbi.nlm.nih.gov/pubmed/?cmd=HistorySearch&querykey=10) | 09:25:38 |
| [#9](http://www.ncbi.nlm.nih.gov/pubmed/advanced) | [Add](http://www.ncbi.nlm.nih.gov/pubmed/advanced) | Search **postoperative morbidity** | [132103](http://www.ncbi.nlm.nih.gov/pubmed/?cmd=HistorySearch&querykey=9) | 09:25:24 |
| [#8](http://www.ncbi.nlm.nih.gov/pubmed/advanced) | [Add](http://www.ncbi.nlm.nih.gov/pubmed/advanced) | Search **postoperative wound complication*** | [245](http://www.ncbi.nlm.nih.gov/pubmed/?cmd=HistorySearch&querykey=8) | 09:25:05 |
| [#7](http://www.ncbi.nlm.nih.gov/pubmed/advanced) | [Add](http://www.ncbi.nlm.nih.gov/pubmed/advanced) | Search **postoperative cardiovascular complication*** | [51](http://www.ncbi.nlm.nih.gov/pubmed/?cmd=HistorySearch&querykey=7) | 09:24:27 |
| [#6](http://www.ncbi.nlm.nih.gov/pubmed/advanced) | [Add](http://www.ncbi.nlm.nih.gov/pubmed/advanced) | Search **postoperative pulmonary complication*** | [989](http://www.ncbi.nlm.nih.gov/pubmed/?cmd=HistorySearch&querykey=6) | 09:24:03 |
| [#5](http://www.ncbi.nlm.nih.gov/pubmed/advanced) | [Add](http://www.ncbi.nlm.nih.gov/pubmed/advanced) | Search **intraoperative complication*** | [31490](http://www.ncbi.nlm.nih.gov/pubmed/?cmd=HistorySearch&querykey=5) | 09:23:36 |
| [#4](http://www.ncbi.nlm.nih.gov/pubmed/advanced) | [Add](http://www.ncbi.nlm.nih.gov/pubmed/advanced) | Search **postoperative complications** | [518093](http://www.ncbi.nlm.nih.gov/pubmed/?cmd=HistorySearch&querykey=4) | 09:23:12 |
| [#3](http://www.ncbi.nlm.nih.gov/pubmed/advanced) | [Add](http://www.ncbi.nlm.nih.gov/pubmed/advanced) | Search **robot-assisted radical cystectomy** | [229](http://www.ncbi.nlm.nih.gov/pubmed/?cmd=HistorySearch&querykey=3) | 09:22:53 |
| [#2](http://www.ncbi.nlm.nih.gov/pubmed/advanced) | [Add](http://www.ncbi.nlm.nih.gov/pubmed/advanced) | Search **open radical cycstectomy** | [5249](http://www.ncbi.nlm.nih.gov/pubmed/?cmd=HistorySearch&querykey=2) | 09:22:23 |
| [#1](http://www.ncbi.nlm.nih.gov/pubmed/advanced) | [Add](http://www.ncbi.nlm.nih.gov/pubmed/advanced) | Search **bladder cancer** | [64441](http://www.ncbi.nlm.nih.gov/pubmed/?cmd=HistorySearch&querykey=1) | 09:21:31 |
